# Estimating the heavy metal concentrations in topsoil in the Daxigou mining area, China, using multispectral satellite imagery

**DOI:** 10.1038/s41598-021-91103-8

**Published:** 2021-06-03

**Authors:** Yun Yang, Qinfang Cui, Peng Jia, Jinbao Liu, Han Bai

**Affiliations:** 1 Key Laboratory of Degraded and Unused Land Consolidation Engineering, The Ministry of Natural Resources, Xi’an, 710075 China; 2 College of Geology Engineering and Geomatics, Chang’an University, Xi’an, 710054 China; 3 KQ GEO Technologies Co., Ltd., Xi’an, 710001 China; 4 Changqing Engineering Design Co., Ltd., Xi’an, 710018 China; 5 Shaanxi Provincial Land Engineering Construction Group Co., Ltd., Xi’an, 710075 China

**Keywords:** Environmental chemistry, Environmental impact

## Abstract

A precise estimation of the heavy metal concentrations in soils using multispectral remote sensing technology is challenging. Herein, Landsat8 imagery, a digital elevation model, and geochemical data derived from soil samples are integrated to improve the accuracy of estimating the Cu, Pb, and As concentrations in topsoil, using the Daxigou mining area in Shaanxi Province, China, as a case study. The relationships between the three heavy metals and soil environmental factors were investigated. The optimal combination of factors associated with the elevated concentrations of each heavy metal was determined combining correlation analysis with collinearity tests. A back propagation network optimised using a genetic algorithm was trained with 80% of the data for samples and subsequently employed to estimate the heavy metal concentrations in the area. The validation results show that the RMSE of the proposed model is lower than those of the existing linear model and rule-based M5 model tree. From the spatial distribution map of the three metals concentrations using the proposed method, there are findings that high concentrations of the heavy metals studied occur in the mining area, across the slag storage area, on the sides of the road used for transporting ore materials, and along the base of slopes in the area. These findings are consistent with the survey results in the field. The validation and findings validate the effectiveness of the proposed method.

## Introduction

Heavy metal pollution of soils around mining areas remains a major concern because of its impacts to the ecological environment and human health^1^. Knowledge on the heavy metal concentrations in soils in mining areas can be exploited for pollution control, ecological protection, and safe-guarding human health. Therefore, accurate determination of heavy metals in soils is currently of significant interest in China. To address this problem, Zhang et al.^2^ built a partial least squares regression model to estimate heavy metals in soils based on soil spectral data measured using an ASD spectrometer. In addition, Gu et al.^3^ employed laser-induced spectroscopy (LIBS) and geochemical data derived from soil samples to estimate heavy metal concentrations in soils around a smelter. However, this site-to-site measurement approach is expensive for large-scale investigations. Therefore, Qu et al.^4^ exploited hyperspectral imagery involving multispectral characteristics to estimate the heavy metal concentrations in soils through regression analysis. Further, Yang et al.^5^ evaluated the feasibility of utilising multiple vegetation indices derived from hyperspectral imagery to estimate the heavy metal concentrations in soils in Yushu County, China. Wang et al.^6^ summarised previous studies on the estimation of heavy metal concentrations in soils based on different data sources and highlighted the challenges and unresolved issues.

Owing to the high cost of acquiring hyperspectral imagery, efforts have been devoted in many studies to assess environmental factors and estimate heavy metal concentrations in soils using multispectral satellite imagery. For example, Peng et al.^7,8^ proposed the use of Landsat 8 imagery to extract spectral indices, in combination with auxiliary data like proximity to road, which were then utilised to establish a model for estimating the heavy metal concentrations in soils. Subsequently, Liu^9^ used Sentinel-2A multispectral imagery to investigate the stress exerted by heavy metals in soils on crops.

According to previous studies, many environmental factors affect the concentrations and spatial distribution of heavy metals in soils^10^, and the interactions among factors such as the soil moisture, clay minerals, metal oxides, and heavy metals have been described^11^.Therefore, determining the optimal combination of environmental factors controlling the accuracy of the estimated heavy metal concentrations is critical. In practice, establishing a high-precision physical model for estimating the heavy metal concentrations in soils is quite challenging.

Therefore, in many studies, shallow machine learning models, such as the random forest algorithm^12^, extreme learning machine^13^ and back propagation (BP) network^14^ have been applied. Compared with deep learning networks, these shallow learning models involve fewer training samples and less time.

Therefore, the estimation of the heavy metal concentrations in soils using remote sensing technology has been significantly advanced by previous studies. However, choosing optimal environmental factors for a precise estimation of the heavy metal concentrations of soils in the mountainous and vegetation-covered mining areas has received little attention. Therefore, in the present study, an approach for accurately estimating the heavy metal concentrations in soils in such areas is investigated for pollution control, ecological protection, and safe-guarding human health.

## Study area and data sources

### Study area

**Figure 1:**
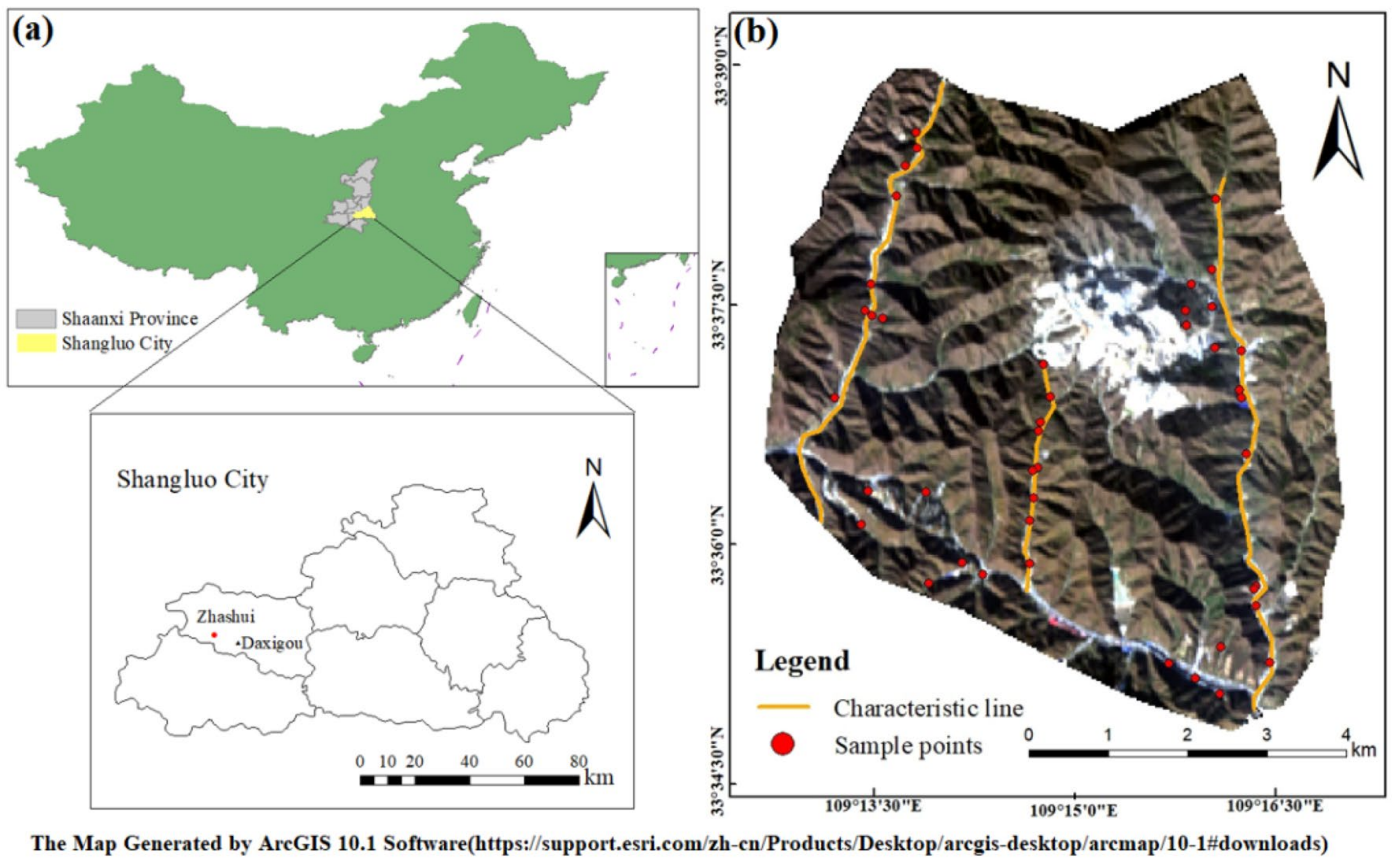
Maps showing (**a**) the location of the Daxigou mining area (enlarged map of Shangluo City) and (**b**) the distribution of sampling sites in a Landsat 8 image involving the spectral bands B4, B3 and B2.

The Daxigou mining area is located in the town of Xiaoling in Zhashui County, Shaanxi Province, China (Fig. 1a). This area hosts the largest siderite deposit in China, which accounts for 47.6% of the total iron ore reserves in Shaanxi Province. In 1982, Northwest Metallurgical Geological Exploration Company, China, reported the abundance of Cu, Pb, As, Ag and other elements in the Daxigou–Yindongzi deposit. In the present study, 39 km^2^ of the Daxigou mining area delineated in Fig. 1b was utilised as the study area. The study area is characterised by elevated Cu and Pb concentrations. This mountainous area involves many intermediate depth and shallow gullies. This area, in combination with significant height differences, represents a complex terrain. Although mining officially began in the area in 1988, open-pit mining was restricted until 2007. The principal land use categories in the area include: mining, cultivation, forest, grass, industrial, and residential. Owing to the long-term mining activities in the area, heavy metal pollution of soils and environmental damage has occurred^15^. Therefore, routine investigations and monitoring of soil heavy metal pollution in the area are necessary.

### Soil sample collection and laboratory analysis

#### Soil sample collection

Forty-four soil samples were collected from the field by technicians in October 2017. Based on the geomorphology and land use in the area, samples were collected to ensure representativeness and uniform distribution along the three main ridges. The sampling sites were mainly in the central portion of the hillsides, which are easily accessible, but near valleys. Heavy metals are commonly transported to the bottom of trenches after scouring, and thus, sampling at the base of slopes was performed at depths of approximately 20–30 cm, while sampling upslope occurred at depths varying between 10 and 20 cm. The distribution of the sampling sites resembled a plum blossom, and each soil samples was collected within a 30 m × 30 m grid of sampling point as the center. Each sample collected comprised a mixture of 1 kg of soils around a sampling location. The geographical coordinates of the area, the soil attributes, and land use categories were documented, and the sampling sites are displayed in Fig. 1b.

#### Physical and chemical analyses of soil samples

The samples were sieved to remove animal and plant remains and dried and crushed before analysis. To determine the heavy metals of interest in the area, a composite soil sample was prepared by taking a little soil from each of the 44 samples. The contents of heavy metals commonly responsible for soil pollution, including Cu, Pb, As, Hg, Cd, Ni, Zn and Cr were determined using conventional methods^16^ by a commercial laboratory in China. Cu and Pb contents were measured using flame atomic absorption spectrophotometry, while the As concentration was determined using the silver diethyldithiocarbamate photometric method. The measured Cu, Pb, As, Hg, Cd, Ni, Zn and Cr concentrations are 68.65, 163.52, 34.8, 0.11, 0.20, 44.4, 130.24 and 35.2 mg kg^–1^, respectively.

The concentrations of the eight elements were compared with background values for the area published in 1990^17^. Based on the differences between the measured and background values as well as the geochemical exploration data for the ore belt, Cu, Pb and As were determined as the heavy metals of interest in the present study.

#### Statistical analysis of the sampling data

A histogram was used to assess the concentrations of each element in all the samples and to remove outliers. Then, all the effective sample data includes 44 samples for Cu, 40 for Pb, and 43 for As. Based on the correlations between the three heavy metals from the least squares regression analysis, each element is examined in more detail subsequently.

### Remote sensing data preparation

Twenty-four Landsat8 images covering the study area that were acquired between 1 January 2017 and 31 December 2017 were retrieved from the US Geological Survey website (https://LPDAAC.usgs.gov); the images affected by cloud cover were excluded. Owing to the lower influence of vegetation from November to March each year, satellite data for this period are more conducive for the assessment of soil properties, and this period was close to that for sample collection. Based on these considerations, in the present study, Landsat 8 images acquired in December 2017 were utilised for estimating the Cu, Pb, and As concentrations in the soils in the study area. The images were corrected for atmospheric effects using the ENVI software, while a digital elevation model (DEM) with a resolution of 30 m for the area was obtained from the geospatial data cloud website (http://www.gscloud.cn/).

## Methodology

### Spectral and terrain factors

#### Spectral reflectance factors

According to previous studies^18^, soils contaminated by heavy metals display spectral characteristics that differ from those of uncontaminated soils. In the present study, the Landsat 8 images were characterised by strong absorption between 400 and 500 nm. The spectral reflectance showed an increasing trend between 500 and 780 nm and a decreasing trend between 780 and 900 nm; the reflectance of images associated with polluted soils exhibited an increasing trend in the range of 1200–2500 nm. These results suggested that these four spectral ranges were suitable for distinguishing heavy metal-contaminated and uncontaminated soils. In this paper, the reflectance of the B2–B7 bands displayed strong correlations with the Pb concentrations of the soils, while those of the B2–B4 bands showed stronger correlations with the Cu and As concentrations. Therefore, based on the geomorphic characteristics of the study area, the spectral values of the B2–B7 bands from the Landsat 8 images were selected. The wavelength ranges for the B2–B7 bands are as follows: B2 (450–510 nm), B3 (530–590 nm), B4 (640–670 nm), B5 (850–880 nm), B6 (1570–1650 nm) and B7 (2110–2290 nm).

#### Spectral index factors

Considering that soil comprises of a mixture of components, the heavy metal content is usually low. Therefore, their characteristics are commonly very weak, which makes it difficult to estimate the heavy metal concentration of soils directly from their spectra, as reported previously^19–21^. However, these concentrations can be obtained indirectly from the adsorption or occurrence relationships between the soil water content, clay mineral content, and environmental factors reflecting vegetation growth, terrain, etc.

Consequently, eight spectral indices reflecting the soil properties associated with heavy metals were derived from the spectral values of the B2–B7 bands of the Landsat 8 images, and these are presented in Table1. The clay mineral ratio (CMR)^22^ is used to highlight the clay mineral content of the soil, and it can indirectly affect the distribution of heavy metals in the soils. The improved normalised water index (MNDWI)^23^ enhances the estimation of the soil water content using satellite images. In an area covered by vegetation, the vegetation growth conditions represented by the normalised vegetation index (NDVI), differential vegetation index (DVI), and enhanced vegetation index (EVI)^24^ can indirectly highlight the soil type^25^ and heavy metal concentrations^26^. In addition, the greenness, brightness, and humidity components generated by the tasselled cap transformation^27^ discriminate the vegetation and soil information.

**Table 1:** Spectral indices used for assessment of the concentrations of Cu, Pb, and As in the soils.

Type	Factors	Definition
Spectral indices	MNDWI	$\left(B3-B6\right)/\left(B3+B6\right)$
	DVI	$\mathrm{B}5/\mathrm{B}4$
	CMR	$B6/B7$
	EVI	$2.5\times \left(B5-B4\right)/\left(B5+6\times B4-7.5\times B2+1\right)$
	NDVI	$\left(B5-B4\right)/\left(B5+B4\right)$
	Greenness	$-0.294\times \mathrm{B}2-0.243\times \mathrm{B}3-0.542\times \mathrm{B}4+0.728\times \mathrm{B}5+0.071\times \mathrm{B}6-0.161\times \mathrm{B}7$
	Brightness	$0.303\times \mathrm{B}2+0.279\times \mathrm{B}3+0.473\times \mathrm{B}4+0.56\times \mathrm{B}5+0.508\times \mathrm{B}6+0.187\times \mathrm{B}7$
	Wetness	$0.151\times \mathrm{B}2+0.197\times \mathrm{B}3+0.328\times \mathrm{B}4+0.341\times \mathrm{B}5-0.712\times \mathrm{B}6-0.456\times \mathrm{B}7$

The indices in Table 1 enhance the spectral characteristics associated with the heavy metal concentrations in soils, and thus, are better for estimating the heavy metal concentrations than the spectral reflectance data provided by the Landsat 8 imagery.

#### Terrain factors

Terrain is also reported to significantly affect the spatial distribution of heavy metals in soils^28^. In the study area, the mountains are partially covered by vegetation, with prominent height differences and steep slopes, which promote the downward movement of heavy metals in the soils around the mining area located in the elevated areas. In addition, the slope direction influences the vegetation growth conditions by, for example, inhibiting the transport of heavy metals in soils towards the base of the mountains by runoff. Therefore, terrain factors impact the spatial distribution of heavy metals in soils. In the present study, altitude, slope, and aspect were utilised in evaluating the spatial distribution of heavy metals in the soils.

### Analysis of environmental factors and optimization

#### Correlations between spectral and terrain factors

The correlations between the concentrations of the three heavy metals and six spectral bands as well as eight spectral indices based on least squares regression are presented in Table 2.

**Table 2:** Correlation coefficients between the three heavy metals and different spectral factors.

Type	Factors	Cu	Pb	As
Spectral reflectance	B2	0.518**	0.419**	0.184
	B3	0.466**	0.418**	0.154
	B4	0.363**	0.428**	0.115
	B5	0.005	0.288	– 0.065
	B6	– 0.088	0.313*	– 0.080
	B7	0.023	0.332*	– 0.038
Spectral indices	DVI	– 0.251	0.115	– 0.173
	EVI	– 0.364*	– 0.286*	– 0.095
	CMR	– 0.453**	– 0.024	– 0.221
	NDVI	– 0.371*	– 0.001	– 0.276
	MNDWI	0.396**	– 0.169	0.297
	Brightness	0.074	0.354*	– 0.022
	Greenness	– 0.386**	– 0.001	– 0.215
	Wetness	0.207	– 0.265	0.120

In Table 2, * represents the significance level at P < 0.05, while ** denotes the significance level at P < 0.01. The results in Table 2 reveal significant correlations between the concentrations of Cu and the spectral reflectance values of the B2, B3, and B4 bands, as well as the values of the CMR, MNDWI, Greenness, EVI, and NDVI. The Pb concentrations are also significantly correlated with the reflectance values of the B2, B3, and B4 bands as well as the EVI and brightness factors. The concentrations of As display less significant correlations with spectral factors, such as the CMR, MNDWI, and NDVI because of less amount of As in soils of the studied area. Therefore, the six spectral bands and eight spectral indices in Table 2 were employed as parameters for estimating the Cu, Pb, and As contents of soils in the area.

To assess the influence of terrain on the spatial distribution of heavy metals in soils in the area, the relationships between terrain factors (altitude, slope, and aspect) and the heavy metals(Cu, Pb and As) were investigated. The metals showed weak correlations (especially Pb and As) with the slope factors. However, by simply considering the correlation coefficients for the study area, the utility of terrain factors in estimating the heavy metal contents in soils cannot be adequately evaluated.

Therefore, in the present study, a comparison of the linear regression before and after the incorporation of terrain factors was conducted. Multivariate linear regression analyses (termed Model I) of the fourteen spectral factors versus the concentrations of the three heavy metals were conducted. In addition, the three terrain factors were incorporated into Model I to produce a new model (Model II), and the regression scores obtained for Models I and II are presented in Table 3.

**Table 3:** Comparison of the linear regression before and after incorporating terrain factors in estimating the heavy metal contents.

	Model	*R*^2^	*P*	*RMSE*
Cu	Model I	0.41	0.00	42.08
	Model II	0.59	0.00	29.87
Pb	Model I	0.29	0.04	25.81
	Model II	0.35	0.03	24.51
As	Model I	0.28	0.00	20.55
	Model II	0.42	0.00	18.48

In general, the coefficients of determination (*R*^2^) for Model II are higher than those for Model I, while the RMSE values for Model II are lower than those for Model I for the three heavy metals. These results indicate that the accuracy of the linear model involving both spectral and terrain factors is superior to that without terrain factors in estimating the concentrations of the three heavy metals in the soils studied. This validates the integration of terrain and spectral factors as a better estimation method. Therefore, subsequently, terrain factors were considered as indicators of the Cu, Pb, and As concentrations in the soils.

#### Optimal factors determination

To determine the optimal factors associated with the concentrations of the three heavy metals in the soils from the 14 spectral and 3 terrain factors, collinearity tests were performed. According to proposed criteria^29^, the collinearity between two factors is weak if their variance expansion factor (VIF) is < 10, and the tested factor is considered as an optimal factor. The optimal factors for Cu, Pb, and As obtained based on this approach are presented in Table 4.

**Table 4:** Correlation and VIF values highlighting the optimal factors associated with the concentrations of Cu, Pb, and As in the soils.

Cu			Pb			As		
Factors	Correlation coefficients	VIF	Factors	Correlation coefficients	VIF	Factors	Correlation coefficients	VIF
Aspect	0.023	1.145	B2	0.419**	3.337	Aspect	– 0.050	1.318
DEM	– 0.084	1.024	Aspect	– 0.262	1.295	DEM	– 0.021	1.344
DVI	– 0.251	8.420	DEM	– 0.179	1.324	Slope	– 0.156	1.841
EVI	– 0.364*	2.485	Slope	– 0.186	1.810	EVI	– 0.095	3.219
CMR	– 0.453**	3.660	EVI	– 0.286*	2.751	CMR	– 0.221	5.494
MNDWI	0.396**	9.318	CMR	– 0.024	5.638	MNDWI	0.297	7.268
			MNDWI	– 0.169	2.718	Brightness	– 0.022	4.901

*represents the significance level at P < 0.05, **denotes the significance level at P < 0.01.

### Genetic algorithm–back propagation model construction

#### Traditional BP network

In contrast to machine learning regression models such as the random forest algorithm, the back propagation (BP) network^30^ and the M5 model tree^14^ are advantageous because of the associated low training cost and universal applicability. Compared with other networks, the BP network is a multilayer feed-forward type suitable for solving complex nonlinear problems. It involves a signal forward transmission and an error back propagation structure. Weights are dynamically adjusted, and the error is estimated by the BP algorithm. However, the random weights and thresholds adopted in the original BP network commonly produce local optimal solutions during gradient descent^30^.

#### Proposed GA–BP model construction

In the present study, a genetic algorithm (GA) for optimising the initial weights and thresholds of the conventional BP model to improve the estimation accuracy and stability is introduced. The steps involved in establishing this GA–BP model are summarised as follows:

1. Initially, the BP network structure was determined. In the present study, a three-layer network comprising the input, hidden, and output was established. The neurons in the input layer were equal to the optimal factors for each metal, whereas the output layer involved just one neuron. In the hidden layer, 4 neurons were assigned randomly, while the Tansig function served as the transfer function.
2. The population of the genetic algorithm was then initialised, and the BP network was trained using training samples. The absolute error between the estimated and the expected output values was considered as the individual fitness value, and this enabled the calculation of the population fitness value. Iterations of both the evolution and the size of the population of the genetic algorithm were set to 15, and the roulette method was used to select the best. The probability of the mutation operation was set to 0.1, and at a crossover probability of 0.3, a real crossover operation was performed. Finally, the optimal individuals obtained from the GA were assigned to the initial weights and the interlayer thresholds of the BP network.
3. In the BP network training using the Levenberg Marquardt nonlinear least squares algorithm, the initial weights and interlayer thresholds were updated according to the error size until the expected error was obtained. The maximum training iterations was 500, with a training accuracy of 0.0001 and learning rate of 0.01. Finally, the GA–BP network was trained using 80% of the data for samples after a random selection (35, 32, and 34 samples for Cu, Pb and As, respectively).

## Results

**Figure 2:**
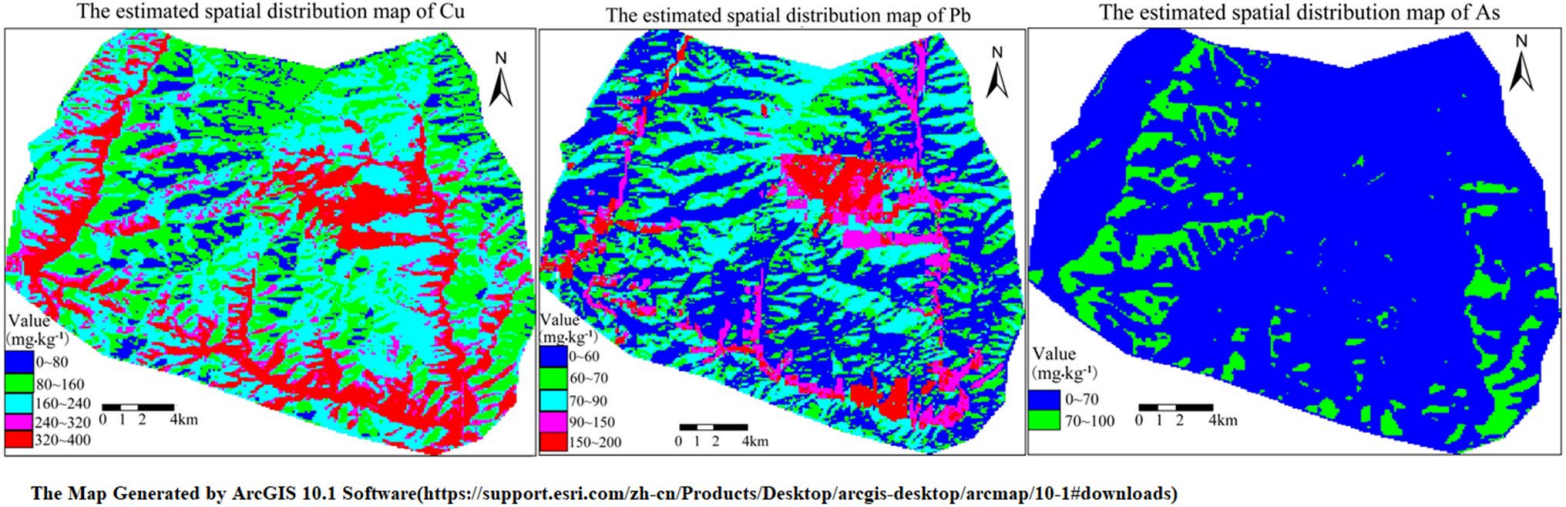
Spatial distribution maps of the concentrations of (**a**) Cu, (**b**) Pb, and (**c**) As in soils in the study area.

Spatial distributions of the three heavy metals in soils in the study area based on the GA–BP model are displayed in Fig. 2.

The data in Fig. 2 reveal that the highest concentrations of the three heavy metals mainly occur in the mining area, the slag stacking area, and the sides of the associated road of the study area. According to the field survey, the ore material is routinely transported from the mining area upslope to roads in the valley. Therefore, because of the accumulation of fallen ore material, all the three metals exhibit high concentrations along roads. The concentrations of Cu, Pb, and As in the study area were evaluated, and the results are presented in Table 5.

**Table 5:** Estimated concentrations of Cu, Pb, and As in soils in the study area.

Cu	Concentration (mg kg^–1^)	0–50	50–180	180–300	300–350	350–400
	Percent (%)	0	70.3	10.1	18.3	1.3
Pb	Concentration (mg kg^–1^)	0–50	50–70	70–90	90–150	150–200
	Percent (%)	42.8	31.5	20.2	3.4	2.1
As	Concentration (mg kg^–1^)	0–40	40–60	60–70	70–80	80–100
	Percent (%)	0	73.5	15.2	5.4	5.1

The data in Table 5 reveal the following: (1) The Cu concentrations in the study area vary between 0 and 400 mg kg^–1^, and 70% of the area involves Cu concentrations ranging between 50 and 180 mg kg^–1^. Pb concentrations ranging between 0 and 200 mg kg^–1^ exist in 94.5% of the area, whereas As concentrations vary between 40 and 100 mg kg^–1^, with concentrations of 40–70 mg kg^–1^ in 88.7% of the area. (2) Cu exhibits the highest concentration, followed by Pb, with As yielding the lowest concentrations in soils in the area. These results are consistent with the geochemical data previously reported^31^. Notably, the concentrations of the three elements in some areas exceed the average values published by the Environmental Protection Administration of China in 1990.

## Discussion and conclusions

To evaluate the estimation error of our model, 20% of the data for the samples unused in the training (9, 8 and 9 for Cu, Pb and As, respectively) were employed for the root mean square error (RMSE) and mean relative estimation error (MRE) calculation using Eqs. (1) and (2), given as follows:

1 $$RMSE=\sqrt{\frac{{\sum }_{i=1}^{N}\left({M}_{i}-{P}_{i}\right)}{N-1}}$$

2 $$MRE=\frac{{\sum }_{i=1}^{N}\left(\frac{{|M}_{i}-{P}_{i}|}{{M}_{i}}\right)}{N}$$

where *M*_*i*_ and *P*_*i*_ represent the measured and estimated values of the heavy metal concentrations of the *i*th test sample, respectively, and *n* denotes the number of test samples. The RMSE and MRE correspondingly reflect the accuracy and average reliability of the estimates from a given model.

To further evaluate the effectiveness of the GA–BP model in improving the estimation accuracy, RMSE and MRE values were also obtained using a multivariate linear model and an M5 model tree^14^ for comparison, and the results are presented in Table 6.

**Table 6:** Comparison of the estimation errors for Cu, Pb, and As based on three models.

	Cu			Pb			As		
	Linear model	M5 model tree	Proposed model	Linear model	M5 model tree	Proposed model	Linear mode	M5 model tree	Proposed model
RMSE	15.338	21.711	6.395	9.703	18.496	4.767	2.266	14.610	1.661
MRE	2.229	0.073	0.025	0.971	0.038	0.060	0.361	0.212	0.309

The RMSE values obtained using the proposed model are 58.30%, 50.87% and 11.77% lower than those obtained from the linear model for Cu, Pb, and As, respectively. The values shown by the proposed model are also lower than those generated using the M5 model tree. Further, the RMSE value for Cu concentrations estimated using the proposed model is higher than those for the other two metals. However, the estimated Cu concentrations using the random forest model also produced an RMSE value of 15.698, which is significantly higher than the value of 6.395 obtained using our model.

**Figure 3:**
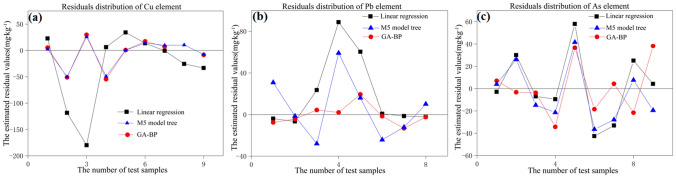
Residual distributions of (**a**) Cu (**b**) Pb and (**c**) As based on the three models.

The estimated residual errors of test samples of Cu, Pb, and As from the three models are shown in Fig. 3. The residual errors based on the GA–BP model are almost 0, and the residual errors for Cu and Pb are significantly lower than those of the linear model and the M5 model tree.

These results demonstrate that the GA-BP model improves the accuracy of estimating the three metals in soils in the study area, and the results are more reliable compared to those of the linear model and the M5 model tree.

Considering the Daxigou mining area in Shaanxi Province, China, as an example, several spectral and terrain factors were integrated in the present study to establish a GA–BP model. The proposed model produced spatial estimates of the concentrations of Cu, Pb, and As in soils in the study area. The main findings of the present study are summarised as follows: (1) Compared with the linear model and the M5 model tree, the proposed GA–BP model improved the accuracy of estimating the Cu, Pb, and As concentrations in soils in the study area. (2) Cu yielded the highest concentration in the soils, followed by Pb and As, and these results are consistent with the geochemical data for the area reported in 1982.The concentrations of the three metals in some soils in the area exceeded the reported background values. (3) The highest concentrations of the three metals occurred near the mining area, across the slag storage area, along road sides, and at the base of the slopes. The spatial distribution produced using the proposed model was also consistent with the field survey results, and thus, validated its effectiveness.

However, the present study is limited, thermal infrared bands of remote sensing imagery will be exploited for additional indices in the future. In addition, the relationships between organic matter, metal oxides, and heavy metals in soils will be considered to highlight the most effective indicators.
